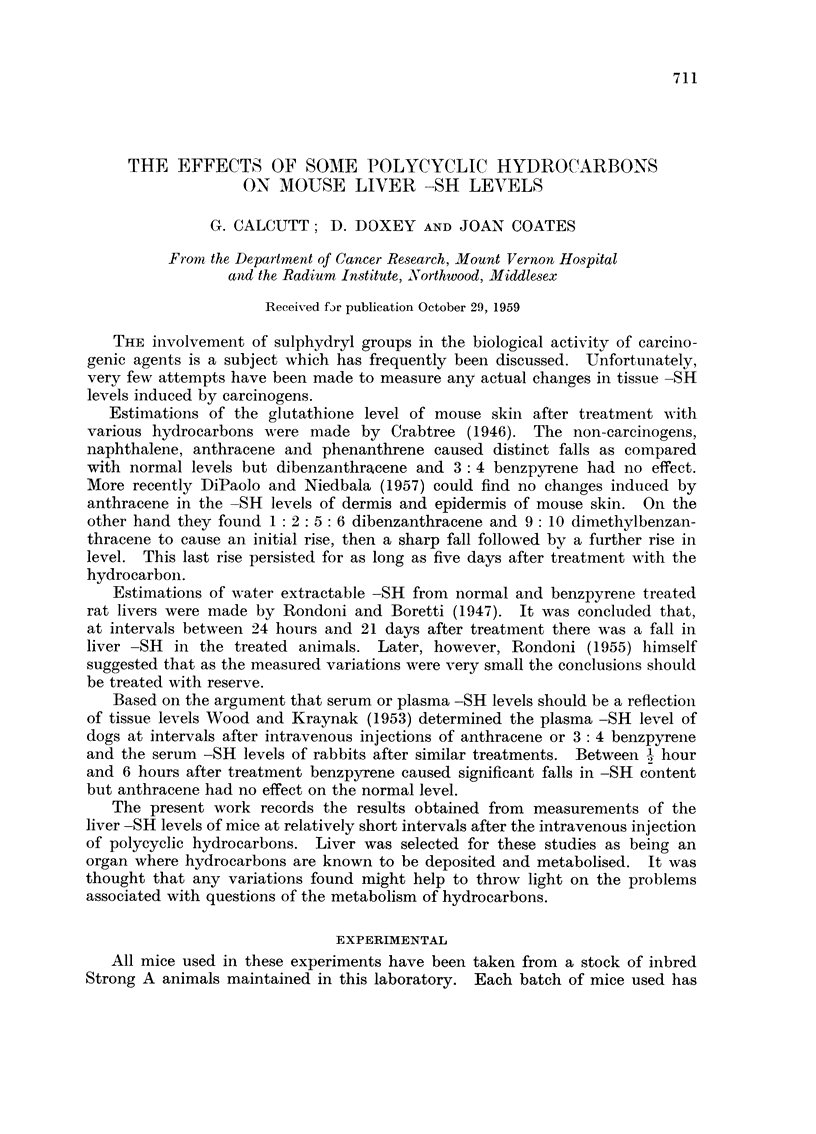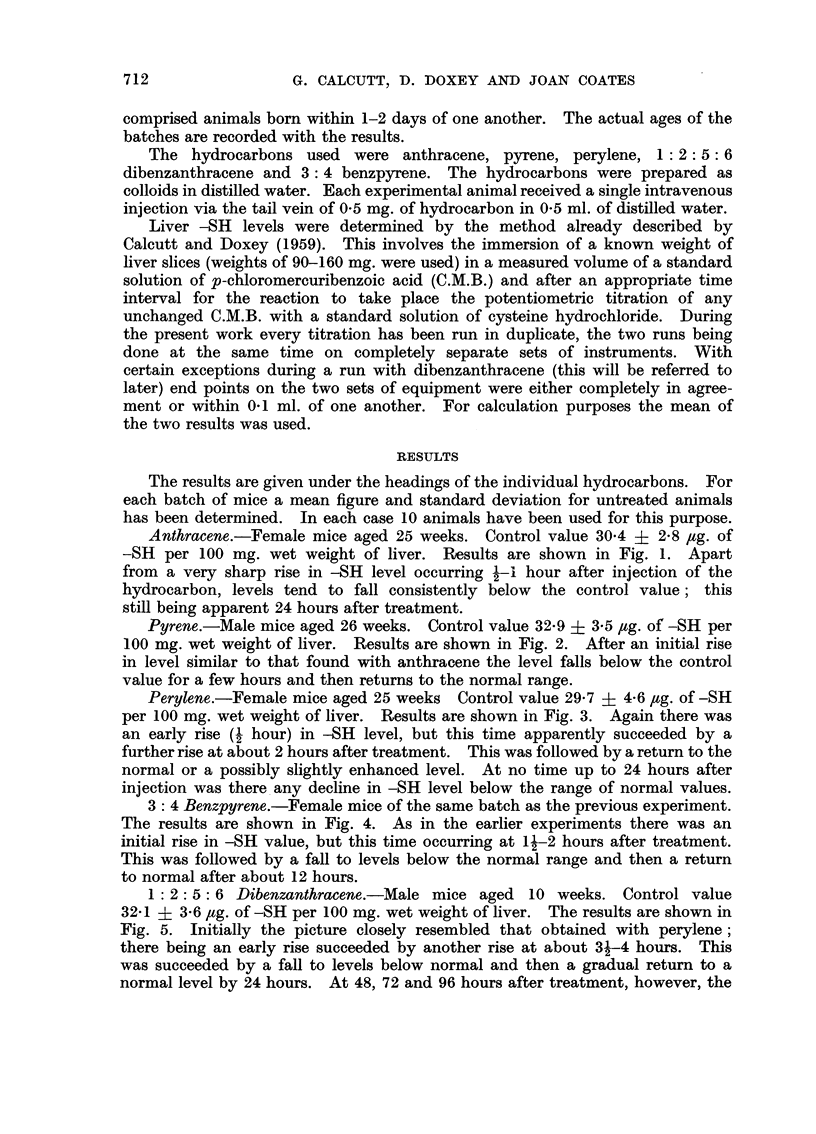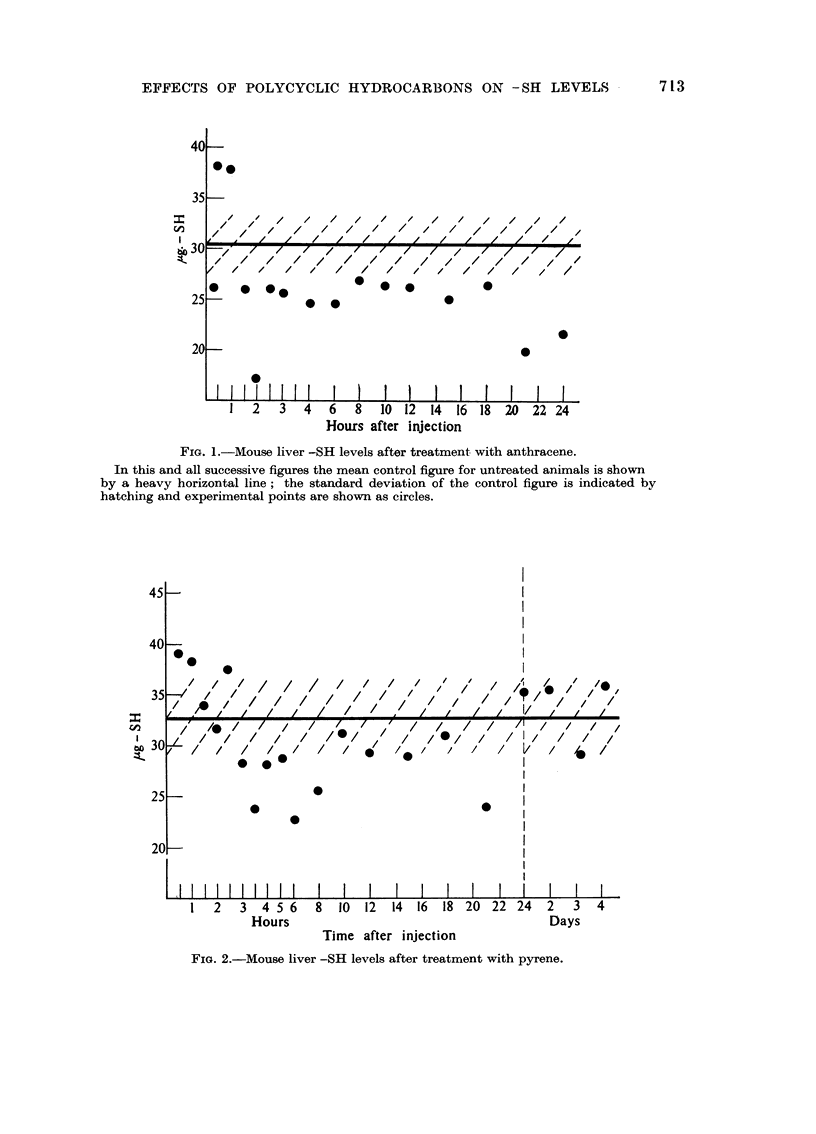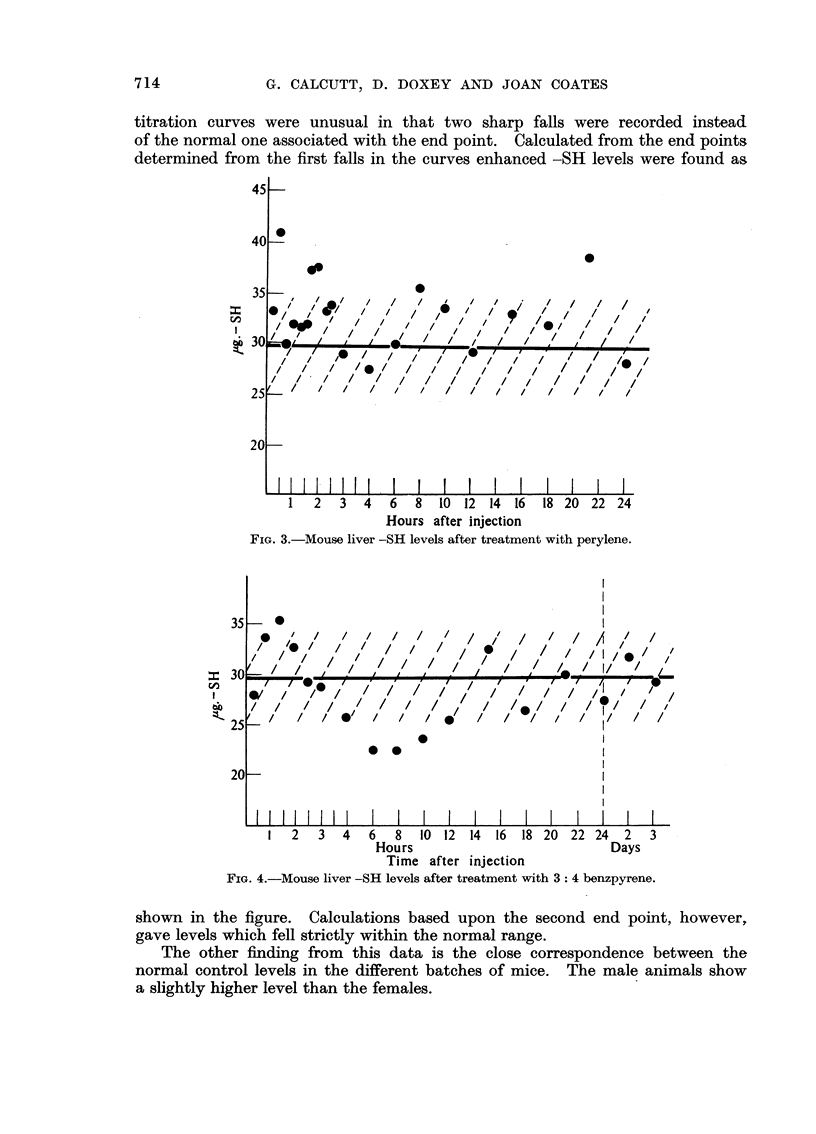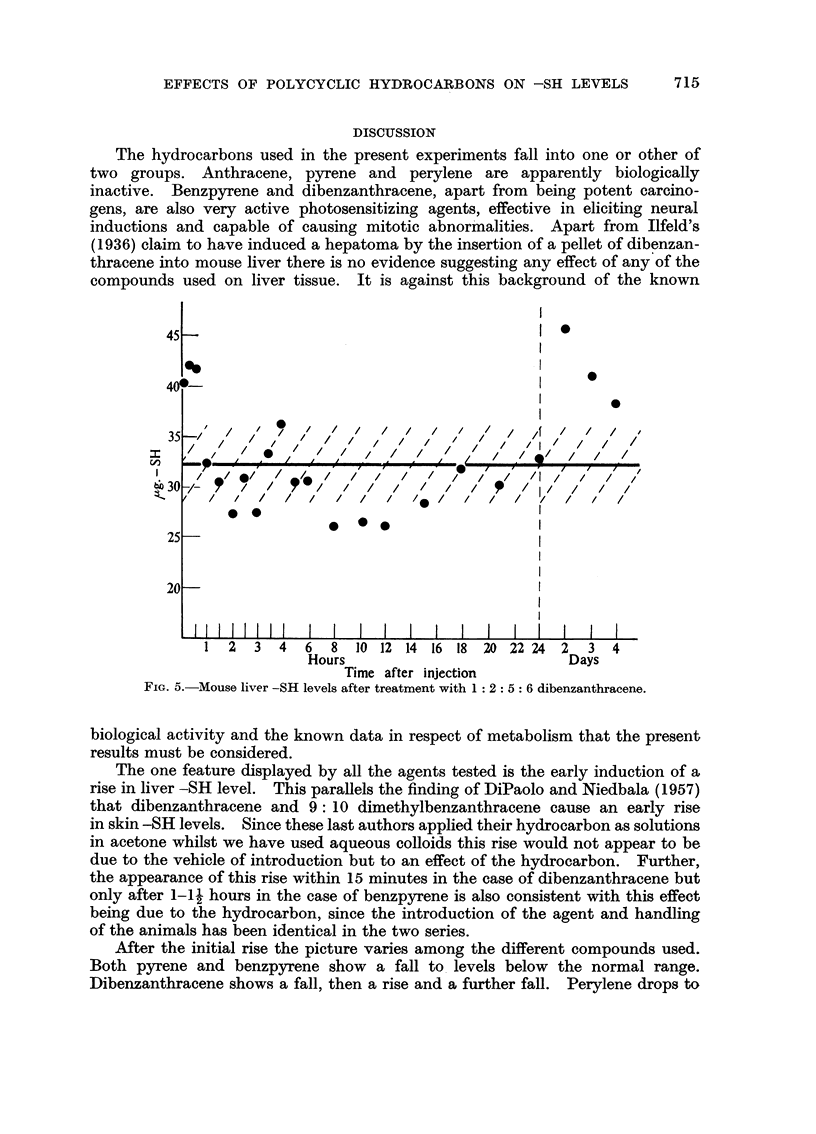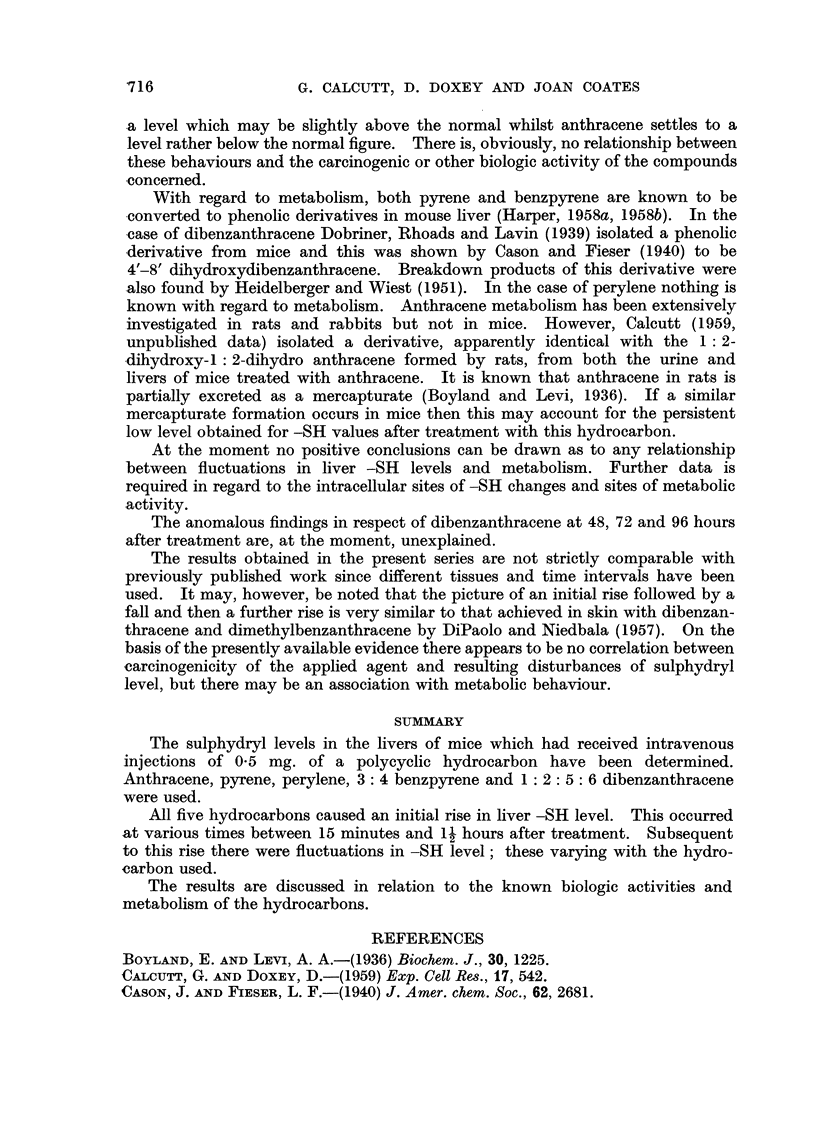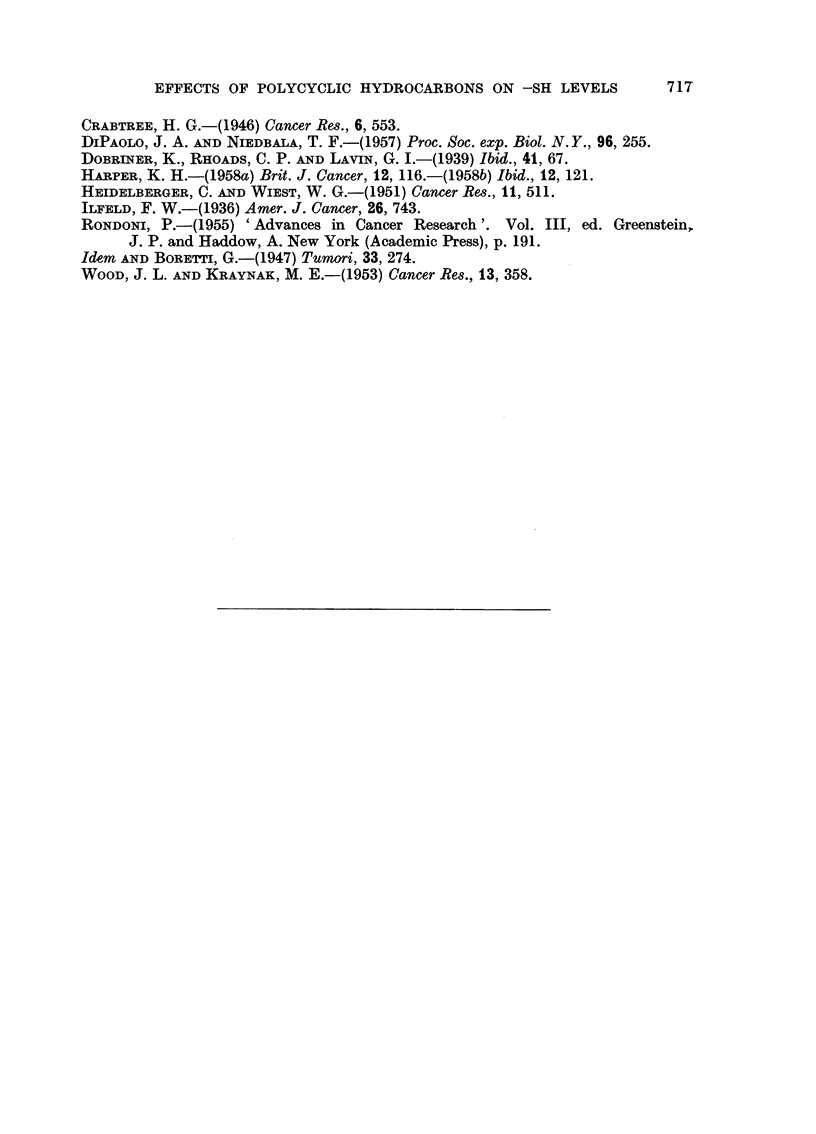# The Effects of some Polycyclic Hydrocarbons on Mouse Liver -SH Levels

**DOI:** 10.1038/bjc.1959.80

**Published:** 1959-12

**Authors:** G. Calcutt, D. Doxey, Joan Coates


### THE EFFECTS OF SOME POLYCYCLIC HYDROCARBONS ON MOUSE LIVER –SH LEVELS

G. CALCUTT ; D. DOXEY AND JOAN COATES

*From the Department of Cancer Research, Mount Vernon Hospital and the Radium Institute, Northwood, Middlesex*

Received for publication October 29, 1959

THE involvement of sulphydryl groups in the biological activity of carcinogenic agents is a subject which has frequently been discussed. Unfortunately, very few attempts have been made to measure any actual changes in tissue –SH levels induced by carcinogens.

Estimations of the glutathione level of mouse skin after treatment with various hydrocarbons were made by Crabtree (1946). The non-carcinogens, naphthalene, anthracene and phenanthrene caused distinct falls as compared with normal levels but dibenzanthracene and 3 : 4 benzpyrene had no effect. More recently DiPaolo and Niedbala (1957) could find no changes induced by anthracene in the –SH levels of dermis and epidermis of mouse skin. On the other hand they found 1 : 2 : 5 : 6 dibenzanthracene and 9 : 10 dimethylbenzanthracene to cause an initial rise, then a sharp fall followed by a further rise in level. This last rise persisted for as long as five days after treatment with the hydrocarbon.

Estimations of water extractable –SH from normal and benzpyrene treated rat livers were made by Rondoni and Boretti (1947). It was concluded that, at intervals between 24 hours and 21 days after treatment there was a fall in liver –SH in the treated animals. Later, however, Rondoni (1955) himself suggested that as the measured variations were very small the conclusions should be treated with reserve.

Based on the argument that serum or plasma –SH levels should be a reflection of tissue levels Wood and Kraynak (1953) determined the plasma –SH level of dogs at intervals after intravenous injections of anthracene or 3 : 4 benzpyrene and the serum –SH levels of rabbits after similar treatments. Between $\frac{1}{2}$ hour and 6 hours after treatment benzpyrene caused significant falls in –SH content but anthracene had no effect on the normal level.

The present work records the results obtained from measurements of the liver –SH levels of mice at relatively short intervals after the intravenous injection of polycyclic hydrocarbons. Liver was selected for these studies as being an organ where hydrocarbons are known to be deposited and metabolised. It was thought that any variations found might help to throw light on the problems associated with questions of the metabolism of hydrocarbons.

### EXPERIMENTAL

All mice used in these experiments have been taken from a stock of inbred Strong A animals maintained in this laboratory. Each batch of mice used has comprised animals born within 1–2 days of one another. The actual ages of the batches are recorded with the results.

The hydrocarbons used were anthracene, pyrene, perylene, 1 : 2 : 5 : 6 dibenzanthracene and 3 : 4 benzpyrene. The hydrocarbons were prepared as colloids in distilled water. Each experimental animal received a single intravenous injection via the tail vein of 0·5 mg. of hydrocarbon in 0·5 ml. of distilled water.

Liver –SH levels were determined by the method already described by Calcutt and Doxey (1959). This involves the immersion of a known weight of liver slices (weights of 90–160 mg. were used) in a measured volume of a standard solution of *p*-chloromercuribenzoic acid (C.M.B.) and after an appropriate time interval for the reaction to take place the potentiometric titration of any unchanged C.M.B. with a standard solution of cysteine hydrochloride. During the present work every titration has been run in duplicate, the two runs being done at the same time on completely separate sets of instruments. With certain exceptions during a run with dibenzanthracene (this will be referred to later) end points on the two sets of equipment were either completely in agreement or within 0·1 ml. of one another. For calculation purposes the mean of the two results was used.

### RESULTS

The results are given under the headings of the individual hydrocarbons. For each batch of mice a mean figure and standard deviation for untreated animals has been determined. In each case 10 animals have been used for this purpose.

*Anthracene.*—Female mice aged 25 weeks. Control value 30·4 $\pm$ 2·8 $\mu$g. of –SH per 100 mg. wet weight of liver. Results are shown in Fig. 1. Apart from a very sharp rise in –SH level occurring $\frac{1}{2}$–1 hour after injection of the hydrocarbon, levels tend to fall consistently below the control value; this still being apparent 24 hours after treatment.

*Pyrene.*—Male mice aged 26 weeks. Control value 32·9 $\pm$ 3·5 $\mu$g. of –SH per 100 mg. wet weight of liver. Results are shown in Fig. 2. After an initial rise in level similar to that found with anthracene the level falls below the control value for a few hours and then returns to the normal range.

*Perylene.*—Female mice aged 25 weeks Control value 29·7 $\pm$ 4·6 $\mu$g. of –SH per 100 mg. wet weight of liver. Results are shown in Fig. 3. Again there was an early rise ($\frac{1}{2}$ hour) in –SH level, but this time apparently succeeded by a further rise at about 2 hours after treatment. This was followed by a return to the normal or a possibly slightly enhanced level. At no time up to 24 hours after injection was there any decline in –SH level below the range of normal values.

3 : 4 *Benzpyrene.*—Female mice of the same batch as the previous experiment. The results are shown in Fig. 4. As in the earlier experiments there was an initial rise in –SH value, but this time occurring at 1$\frac{1}{2}$–2 hours after treatment. This was followed by a fall to levels below the normal range and then a return to normal after about 12 hours.

1 : 2 : 5 : 6 *Dibenzanthracene.*—Male mice aged 10 weeks. Control value 32·1 $\pm$ 3·6 $\mu$g. of –SH per 100 mg. wet weight of liver. The results are shown in Fig. 5. Initially the picture closely resembled that obtained with perylene; there being an early rise succeeded by another rise at about 3$\frac{1}{2}$–4 hours. This was succeeded by a fall to levels below normal and then a gradual return to a normal level by 24 hours. At 48, 72 and 96 hours after treatment, however, the

FIG. 1.—Mouse liver -SH levels after treatment with anthracene.

In this and all successive figures the mean control figure for untreated animals is shown by a heavy horizontal line ; the standard deviation of the control figure is indicated by hatching and experimental points are shown as circles.

FIG. 2.—Mouse liver -SH levels after treatment with pyrene.

titration curves were unusual in that two sharp falls were recorded instead of the normal one associated with the end point. Calculated from the end points determined from the first falls in the curves enhanced –SH levels were found as

FIG. 3.—Mouse liver –SH levels after treatment with perylene.

FIG. 4.—Mouse liver –SH levels after treatment with 3 : 4 benzpyrene.

shown in the figure. Calculations based upon the second end point, however, gave levels which fell strictly within the normal range.

The other finding from this data is the close correspondence between the normal control levels in the different batches of mice. The male animals show a slightly higher level than the females.

### DISCUSSION

The hydrocarbons used in the present experiments fall into one or other of two groups. Anthracene, pyrene and perylene are apparently biologically inactive. Benzpyrene and dibenzanthracene, apart from being potent carcinogens, are also very active photosensitizing agents, effective in eliciting neural inductions and capable of causing mitotic abnormalities. Apart from Ilfeld's (1936) claim to have induced a hepatoma by the insertion of a pellet of dibenzanthracene into mouse liver there is no evidence suggesting any effect of any of the compounds used on liver tissue. It is against this background of the known

FIG. 5.—Mouse liver –SH levels after treatment with 1 : 2 : 5 : 6 dibenzanthracene.

biological activity and the known data in respect of metabolism that the present results must be considered.

The one feature displayed by all the agents tested is the early induction of a rise in liver –SH level. This parallels the finding of DiPaolo and Niedbala (1957) that dibenzanthracene and 9 : 10 dimethylbenzanthracene cause an early rise in skin –SH levels. Since these last authors applied their hydrocarbon as solutions in acetone whilst we have used aqueous colloids this rise would not appear to be due to the vehicle of introduction but to an effect of the hydrocarbon. Further, the appearance of this rise within 15 minutes in the case of dibenzanthracene but only after 1–1½ hours in the case of benzpyrene is also consistent with this effect being due to the hydrocarbon, since the introduction of the agent and handling of the animals has been identical in the two series.

After the initial rise the picture varies among the different compounds used. Both pyrene and benzpyrene show a fall to levels below the normal range. Dibenzanthracene shows a fall, then a rise and a further fall. Perylene drops to a level which may be slightly above the normal whilst anthracene settles to a level rather below the normal figure. There is, obviously, no relationship between these behaviours and the carcinogenic or other biologic activity of the compounds concerned.

With regard to metabolism, both pyrene and benzpyrene are known to be converted to phenolic derivatives in mouse liver (Harper, 1958*a*, 1958*b*). In the case of dibenzanthracene Dobriner, Rhoads and Lavin (1939) isolated a phenolic derivative from mice and this was shown by Cason and Fieser (1940) to be 4′–8′ dihydroxydibenzanthracene. Breakdown products of this derivative were also found by Heidelberger and Wiest (1951). In the case of perylene nothing is known with regard to metabolism. Anthracene metabolism has been extensively investigated in rats and rabbits but not in mice. However, Calcutt (1959, unpublished data) isolated a derivative, apparently identical with the 1 : 2-dihydroxy-1 : 2-dihydro anthracene formed by rats, from both the urine and livers of mice treated with anthracene. It is known that anthracene in rats is partially excreted as a mercapturate (Boyland and Levi, 1936). If a similar mercapturate formation occurs in mice then this may account for the persistent low level obtained for –SH values after treatment with this hydrocarbon.

At the moment no positive conclusions can be drawn as to any relationship between fluctuations in liver –SH levels and metabolism. Further data is required in regard to the intracellular sites of –SH changes and sites of metabolic activity.

The anomalous findings in respect of dibenzanthracene at 48, 72 and 96 hours after treatment are, at the moment, unexplained.

The results obtained in the present series are not strictly comparable with previously published work since different tissues and time intervals have been used. It may, however, be noted that the picture of an initial rise followed by a fall and then a further rise is very similar to that achieved in skin with dibenzanthracene and dimethylbenzanthracene by DiPaolo and Niedbala (1957). On the basis of the presently available evidence there appears to be no correlation between carcinogenicity of the applied agent and resulting disturbances of sulphydryl level, but there may be an association with metabolic behaviour.

### SUMMARY

The sulphydryl levels in the livers of mice which had received intravenous injections of 0·5 mg. of a polycyclic hydrocarbon have been determined. Anthracene, pyrene, perylene, 3 : 4 benzpyrene and 1 : 2 : 5 : 6 dibenzanthracene were used.

All five hydrocarbons caused an initial rise in liver –SH level. This occurred at various times between 15 minutes and 1½ hours after treatment. Subsequent to this rise there were fluctuations in –SH level ; these varying with the hydrocarbon used.

The results are discussed in relation to the known biologic activities and metabolism of the hydrocarbons.

### REFERENCES

Boyland, E. and Levi, A. A.—(1936) *Biochem. J.*, **30**, 1225.
Calcutt, G. and Doxey, D.—(1959) *Exp. Cell Res.*, **17**, 542.
Cason, J. and Fieser, L. F.—(1940) *J. Amer. chem. Soc.*, **62**, 2681.

CRABTREE, H. G.—(1946) *Cancer Res.*, **6**, 553.
DIPAOLO, J. A. AND NIEDBALA, T. F.—(1957) *Proc. Soc. exp. Biol. N.Y.*, **96**, 255.
DOBRINER, K., RHOADS, C. P. AND LAVIN, G. I.—(1939) *Ibid.*, **41**, 67.
HARPER, K. H.—(1958*a*) *Brit. J. Cancer*, **12**, 116.—(1958*b*) *Ibid.*, **12**, 121.
HEIDELBERGER, C. AND WIEST, W. G.—(1951) *Cancer Res.*, **11**, 511.
ILFELD, F. W.—(1936) *Amer. J. Cancer*, **26**, 743.
RONDONI, P.—(1955) 'Advances in Cancer Research'. Vol. III, ed. Greenstein, J. P. and Haddow, A. New York (Academic Press), p. 191.
*Idem* AND BORETTI, G.—(1947) *Tumori*, **33**, 274.
WOOD, J. L. AND KRAYNAK, M. E.—(1953) *Cancer Res.*, **13**, 358.